# Flood susceptibility assessment using three machine learning techniques and comparison of their performance

**DOI:** 10.1038/s41598-026-38391-0

**Published:** 2026-02-10

**Authors:** Tade Mule Asrade, Sintayehu Adefires Abebe, Kassahun Birhanu Tadesse, Mulu Sewinet Kerebih, Taye Minichil Meshesha

**Affiliations:** 1 Department of Hydraulic and Water Resources Engineering , Debre Markos University , Debre Markos, Ethiopia; 2 School of Engineering Discipline of Agricultural Engineering , University of KwaZulu-Natal , Pietermaritzburg, South Africa

**Keywords:** Choke watershed, Flood susceptibility, Machine learning algorithms, Remote sensing data, Environmental sciences, Hydrology, Natural hazards

## Abstract

One of the most common natural disasters is flooding, which has the potential to seriously harm environments and infrastructure. Flood susceptibility mapping (FSM) is the main way to manage flood risk. It measures how likely a region is to flood in a quantitative way. The purpose of this study was to develop state-of-the-art ensemble machine learning (ML) models for flood prediction and to identify the most suitable approach for accurate flood susceptibility mapping. This study leverages diverse datasets, including elevation, slope, aspect, plan curvature, topographic wetness index, stream power index, distance from rivers, soil, rainfall, land use/land cover, and drainage density, which were used as conditioning factors to evaluate flood susceptibility in the Choke Watershed. Three machine learning (ML) algorithms were employed: Random Forest (RF), Gradient Boosting (GB), and Extreme Gradient Boosting (XGBoost). Model performance was assessed using confusion matrix metrics and the area under the receiver operating characteristic curve (AUROC). The Gradient Boosting (GB) and Extreme Gradient Boosting (XGBoost) models scored the highest in terms of test accuracy (0.97), followed by RF (0.96). This study is the first application of these models in the Choke Watershed for flood susceptibility mapping, with potential for broader applications to other natural disasters, including earthquakes and landslides. The results help strengthen global efforts aimed at mitigating natural disaster risks, particularly in Ethiopia, and advancing environmental sustainability.

## Introduction

Floods are among the most devastating natural disasters globally, responsible for over 5,000 deaths annually and causing widespread socio-economic and environmental damage^1^. Their increasing frequency is driven by both climatic factors—such as intense rainfall, snowmelt, and extreme weather—and anthropogenic pressures like deforestation, rapid urbanization, and poor land-use practices^1–4^.

Flood susceptibility mapping has become a critical tool for disaster risk reduction worldwide, aiding in early warning systems, infrastructure planning, and emergency response^4–6^. Countries like China, India, Australia, and Egypt have adopted machine learning (ML) approaches to enhance flood prediction accuracy^7,8^. In contrast, many Sub-Saharan African nations, including Ethiopia, face challenges in adopting such methods due to limited data availability and technological constraints.

East African cities are particularly vulnerable to flooding, exacerbated by unregulated urban expansion and inadequate early warning systems^9^. Ethiopia, one of the most disaster-prone countries in the region, experienced 86 major natural disasters between 1980 and 2010, with floods ranking second after droughts^10^. Recent events have displaced tens of thousands and damaged infrastructure, farmland, and homes across the Amhara, Oromia, SNNP, Somali, and Dire Dawa regions^11^. Flash floods in areas like Tigray, Wollo, Gojjam, Shewa, Wolayita, and Debre Markos often occur without warning, leaving communities highly vulnerable^12^.

Flood assessments are complex due to the interplay of various climatic and anthropogenic factors. With an ever-growing global population, the frequency and severity of floods have increased, exacerbated by climate change. In regions like Ethiopia, where climate variability is pronounced—ranging from cold winters and heavy snow in the northwest to rapid urban expansion—both climate and human activities contribute significantly to flood occurrence^13^. As flood risks grow under climate change, accurate susceptibility assessments become increasingly vital.

Traditionally, physically-based models^14^ were employed for flood prediction, including storm tracking^15^, rainfall-runoff relationships^16^, and hydraulic flow simulations^17^. While these models excel in predicting a range of hydrological events, they often require extensive datasets and high computational resources, making them unsuitable for short-term flood predictions^16^. Similarly, statistical models such as the Autoregressive Moving Average (ARMA)^18^, multiple linear regression (MLR)^19^, and autoregressive integrated moving average (ARIMA)^20^ are the most common flood frequency analysis (FFA) methods for modeling flood prediction. While these models offer advantages in terms of efficiency, they also have limitations in short-term prediction accuracy, requiring a decade of data for meaningful long-term forecasting^21^. Additionally, they are often unsuitable for use in regions lacking long-term data, necessitating regional flood frequency analyses (RFFA) such as MISBA and Sacramento models^22^ and Sacramento^23^.

To overcome these challenges, machine learning (ML) techniques have emerged as a promising alternative. Machine learning models leverage diverse datasets—including topographic, hydrologic, meteorological, and land use information—to identify patterns and generate flood susceptibility maps. Unlike physically based models, which require extensive computational resources and expert calibration, machine learning approaches offer faster, scalable, and often more accurate predictions. Additionally, they are better equipped to integrate real-time remote sensing data and other geospatial inputs, reducing dependence on field surveys that are often time-consuming and logistically constrained^24–26^.

Among machine learning approaches, ensemble methods such as Random Forest (RF), Gradient Boosting (GB), and Extreme Gradient Boosting (XGBoost) have shown superior performance in flood modeling due to their ability to handle nonlinear relationships and reduce overfitting^27,28^. More recently, comparative analysis of standalone machine learning models, which combine the strengths of multiple algorithms—have gained attention for their enhanced prediction accuracy, reduced bias, and improved generalization. These models outperform single-algorithm approaches by incorporating a broader range of conditioning factors and optimizing variable importance rankings, ultimately reducing misclassification errors in susceptibility mapping^29^.

In Ethiopia, flood risk assessments using geospatial technologies like Remote Sensing (RS) and GIS have been conducted in regions such as the Lower Awash Sub-basin^30^, Kobo Woreda^31^, Gidabo Watershed^32^, the Fetam watershed^33^, and the upper Abay river basin^34^. However, most of these studies rely on traditional modeling approaches and underutilize modern ML techniques.

Recent global studies have successfully implemented machine learning models for flood mapping. For example, Han, et al.^35^ used models like RF, ANN, XGBoost, and GBDT in Jiangxi Province, China, with tree-based models achieving the highest accuracy. Similarly, XGBoost outperformed other classifiers in flash flood prediction in Egypt^36^ and in power load scheduling. However, such models remain underutilized in Ethiopia, particularly in high-risk, ecologically sensitive areas like the Choke watershed.

The Choke watershed—located in East and West Gojjam of the Amhara Region—is a critical sub-basin of the Upper Blue Nile. Known for its biodiversity and hydrological importance, the region is severely affected by seasonal flash floods, exacerbated by deforestation, soil erosion, and population pressure^37^. Notably, a major flood in 2006 highlighted the urgent need for effective flood risk management in the area.

Yet, no high-resolution flood susceptibility mapping using advanced ML techniques has been conducted in the Choke watershed. To address this gap, the present study aims to develop accurate flood susceptibility maps using comparative analysis of standalone machine learning models: Random Forest (RF), Gradient Boosting (GB), and Extreme Gradient Boosting (XGBoost). Eleven flood-conditioning factors—encompassing topographic, hydrologic, soil, and anthropogenic variables—are integrated to identify high-risk zones and support disaster risk management strategies.

This study also compares the predictive performance of the three comparative analysis of standalone machine learning models, which, although widely used globally, have rarely been applied collectively in Ethiopia. To the best of the authors’ knowledge, this is the first research to evaluate and implement all three algorithms in combination for flood susceptibility analysis in the Choke watershed.

The novelty of this research lies in its application of a comparative analysis of standalone machine learning models for flood susceptibility mapping in the Choke watershed an ecologically sensitive and flood-prone area that has not been previously studied using advanced data-driven methods. This study integrates a comprehensive set of eleven flood-conditioning factors, carefully selected to reflect the region’s unique topographical, hydrological, and socio-environmental characteristics. Furthermore, it offers a comparative analysis of three robust machine learning algorithms—Random Forest (RF), Gradient Boosting (GB), and Extreme Gradient Boosting (XGBoost)—to evaluate their performance in data-scarce and topographically complex settings. The findings are expected to enhance understanding of flood dynamics in the Choke watershed, provide a valuable decision-support tool for planners and policymakers, and strengthen Ethiopia’s capacity for disaster risk reduction. Ultimately, this research contributes to broader global efforts toward natural hazard mitigation, sustainable land use, and climate adaptation.

## Materials and methods

### Study area

**Fig. 1:**
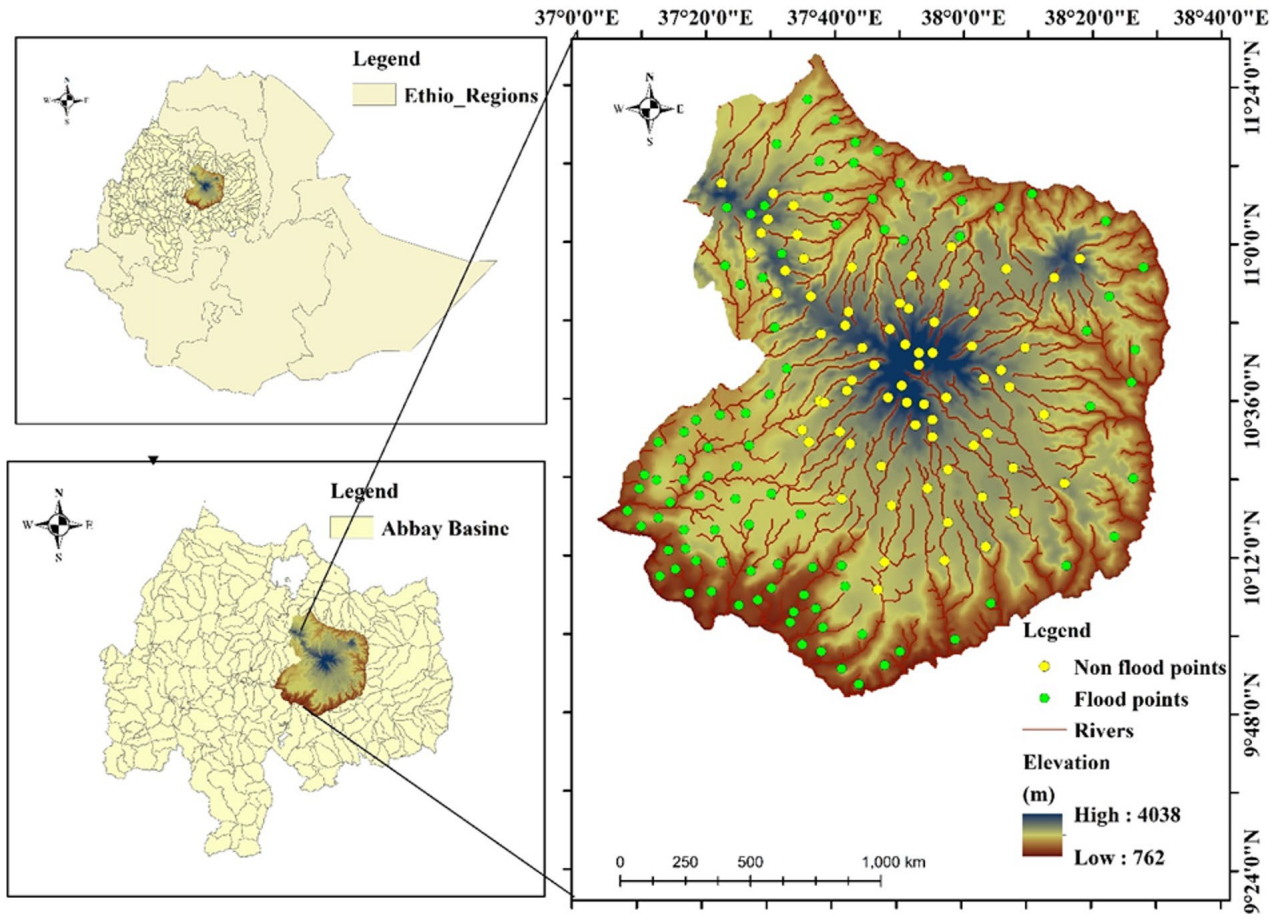
Study area map and flood inventory

Choke is a large block of the highland mountain range, located in the central part of both the East and West Gojjam Administrative Zones in the Amhara National Regional State, northwestern Ethiopia. Choke Mountain serves as a water tower for the upper Blue Nile Basin, acting as the source for over 60 rivers and 270 springs^38^. The catchment area of Choke Mountain spans nine woredas, and the watershed is situated between 9° to 11°N latitude and 37° to 38°E longitude (Fig. 1). Diverse topographical features, ranging from plateaus and deep incised valleys to escarpments, plains, and gorges, characterize the region. Elevations within the area vary from 2,800 to 4,088 m above sea level. Due to the complexity of its topography, the region experiences significant local gradients in precipitation, temperature, and soil properties^39^. Choke Mountain is pivotal to the region’s hydrology, being the primary source of the majority of the tributaries of the Blue Nile River. Among the four major rivers originating from the mountain are the Muga, Chemoga, Abeya, and Techma, in addition to several smaller tributaries (Abay)^39^. The mountain’s abundant water resources support various nature-based tourism activities, including swimming, boating, hiking, and fishing. These water resources are also crucial for other purposes, such as electricity generation, drinking water, irrigation, and washing. Climatically, the Choke Mountain region spans six distinct climatic zones: Upper Kola, Lower Weyna Dega, Upper Weyna Dega, Lower Dega, Upper Dega, and Wurch^40^. Rainfall in the region is highly variable, ranging from 600 to 2,000 mm per year, with local differences due to the topographic gradients^41^. The dominant soil types in the watersheds include Leptosols, Cambisols, Vertisols, Nitosols, Alisols, Luvisols, Andosols, and Phaozems, which support a variety of agricultural activities. Common crops cultivated in the region include sorghum, maize, teff, durum wheat, barley, chickpeas, and various pulses and potatoes, depending on the agro-ecological zone^42^. Maps were created using ArcGIS software (version 10.4; Esri, Redlands, CA, USA; https://www.esri.com*).*

Major flood incidents during the last ten years were compiled from the National Disaster Risk Management Commission (NDRMC), the National Meteorological Agency (NMA), existing research, and remote sensing assessments to put the significance of flood susceptibility assessment into perspective (Table 1). Recurrent flooding in the Choke Watershed has caused agricultural losses, displacement, and infrastructural damage throughout the Kiremt (major rainy) season, which runs from June to September. This recurrent trend emphasizes the necessity of data-driven mapping of the region’s flood susceptibility.

**Table 1:** Major flood events in the choke watershed.

Year	Month/Season	Main affected districts (woredas)	Estimated area inundated (km²)	Reported impacts	Sources
2015–2020	June-August	Debre Markos, Sinan, Wuseta	14–27	Crop losses, damage to rural roads and irrigation structures	Addis et al^34^.
1995–2022	July–August	Ferse Bet, Dega Damot	83	Farmland inundated; households temporarily displaced	Negese et al^43^.
2018	Jun-Sep	Wuseta, Debre markos	46	Flash floods damaged roads and schools; livestock loss	Mulu et al^12^.
2010, 2016, 2012, 2020	July–September	Gozamin, Machakel, Sinan	~ 63	cropland flooded; households displaced; bridge failures	Mamo et al^44^.
2021	August	Dembecha, Hulet Ej Enese	~ 47	Severe flash floods following intense rainfall; loss of property and stored grain	NDRMC, woreda offices
2022	July–August	Gozamin, Bibugn	~ 52	villages affected; teff and maize fields inundated	NDRMC, field survey

### Flood inventory map

Flood susceptibility mapping is a binary classification process that assigns a value of 1 to flood-affected locations and 0 to non-flood-affected locations^4,45^. In this study, a flood inventory map was developed to represent spatially distributed flood and non-flood areas based on five significant flood events that occurred in 2005, 2010, 2013, 2016, and 2020. Data were primarily obtained from the National Disaster Risk Management Commission (NDRMC), Sentinel-1 Synthetic Aperture Radar (SAR) imagery, and complementary hydrological and logistical records^44,46,47^.

For each flood event, pre- and post-flood Sentinel-1 SAR images were mosaicked and processed using speckle filtering to minimize image noise. The inundated areas were identified using a change detection analysis, and then flooded pixels were identified using a 1 (positive) value and non-flooded locations using a 0 (negative) value. This was done using threshold-based classification. To ensure precise delineation of flood extents, a Digital Elevation Model (DEM) was utilized to rectify geometric distortions and eliminate permanent water bodies and high slopes (> 5%)^4,26^.

Ultimately, a total of 300 observation points were identified for the flood inventory map, comprising 210 flood-affected and 90 non-flood-affected locations. To improve dataset representativeness and reduce potential class imbalance, an additional 120 non-flood points were randomly generated in well-drained, flood-free areas, resulting in a balanced dataset of 210 flood and 210 non-flood points for modeling.

The non-flood points were carefully selected from higher elevation, well-drained, and flood-free areas confirmed through DEM-derived slope and elevation data. Their reliability was validated using multi-temporal analysis of Sentinel-1 SAR imagery, Google Earth Pro time-series inspection, and field verification during dry seasons. Additionally, historical flood records (2000–2020) and flood damage reports were reviewed to confirm the absence of inundation. A buffer zone was created around previously flooded areas to avoid selecting non-flood points near transitional or potentially flood-prone zones. This combined approach ensured the credibility and spatial representativeness of the non-flood samples^26,45,48^.

The dataset was divided into 70% for model training and 30% for validation, following established practices in flood susceptibility studies^29,49–51^. This ratio provides an optimal balance between ensuring sufficient data for model training—enabling the algorithms to capture spatial variability and complex feature relationships—and maintaining a robust independent validation subset for performance assessment. Several previous flood-related studies have demonstrated that a 70:30 (or similar 75:25) ratio yields stable and generalizable model results while avoiding overfitting^52^. In this study, the chosen split ensured that the models were trained with an adequately large and diverse dataset while preserving enough data to independently validate predictive accuracy and generalization capability.

**Fig. 2:**
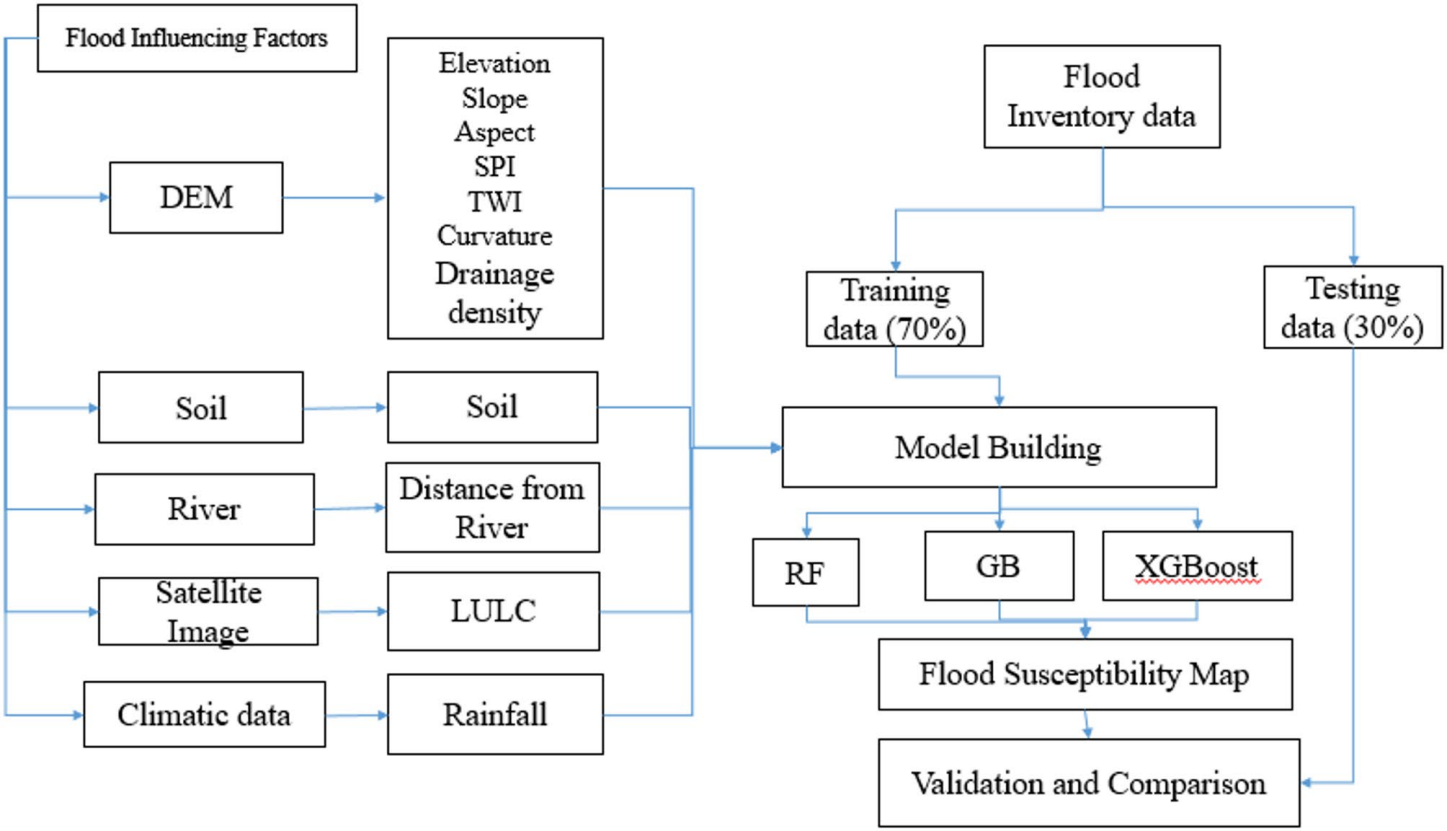
Methodological workflow adopted in this study

The methodological flowchart followed in this study is shown in Fig. 2.

### Flood-influencing factors

Previous studies have identified four primary groups of factors influencing flood occurrence: geomorphological, geological, hydrological and climatic, and anthropogenic activities^53^. Based on these studies and the availability of relevant data in the study area, eleven flood-conditioning factors were selected for analysis. These include elevation, slope, aspect, plan curvature, topographic wetness index (TWI), SPI, drainage density (Dd), distance from river, soil types, land use/land cover (LULC), and rainfall patterns.

Digital Elevation Model (DEM) data were obtained from the obtained from ALOS-PALSAR. From the DEM, key topographic parameters such as elevation, slope, aspect, plan curvature, Dd, and TWI were derived. These parameters are widely used in previous flood susceptibility studies due to their effectiveness in accurately mapping potential flood-prone areas. All spatial data were processed and resampled within a Geographic Information System (GIS) environment; using raster data with a spatial resolution of 30 m (see Table 2 for data sources).

**Table 2:** Data type and sources used in this study.

No.	Flood-conditioning factor	Source	Spatial resolution	Time period	Data type
1	Elevation	Alaska Satellite Facility (ASF): https://www.asf.alaska.edu	30 m	2022	Raster
2	Slope	Derived from DEM	30 m		Raster
3	Curvature	Derived from DEM	30 m		Raster
4	Stream power index (SPI)	Derived from DEM	30 m		Raster
5	Topographic wetness index (TWI)	Derived from DEM	30 m		Raster
6	Aspect	Derived from DEM	30 m		Raster
7	Rainfall	National Meteorological Agency of Ethiopia	30 m	1994–2023	Raster
8	Drainage density	Derived from DEM	30 m		Raster
9	Distance from river	Derived from stream network	30 m		Raster
10	Soil	Harmonized World Soil Database (HWSD)	1 km	2008 (Version 1.2)	Raster
11	Land use/Land cover	USGS–GLOVIS (Landsat-8 imagery)	30 m	2022	Raster

#### Elevation

**Fig. 3:**
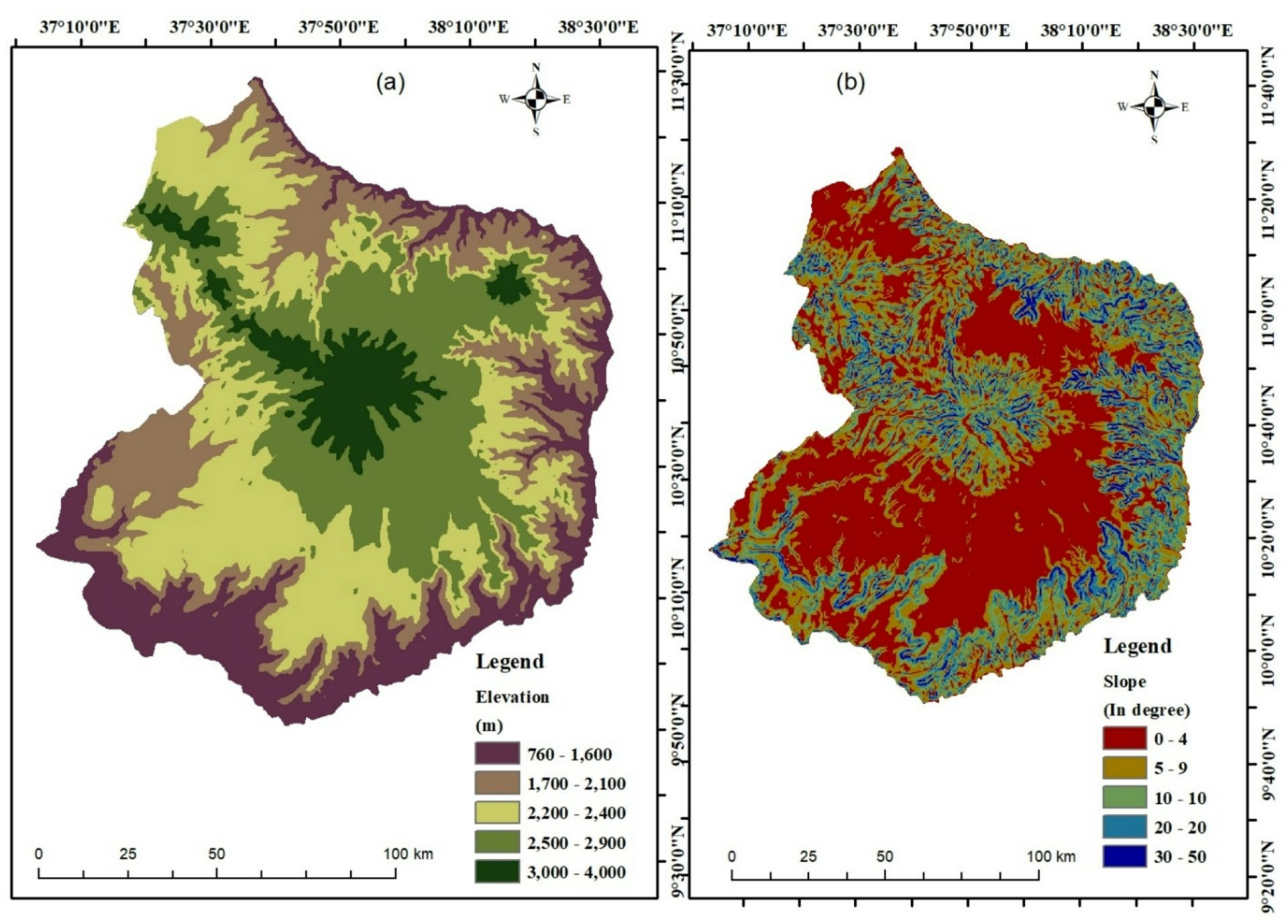
Maps of flood conditioning factors: (**a**) elevation, (**b**) slope, (**c**) aspect, (**d**) curvature, (**e**) land use, (**f**) soil, (**g**) topographic wetness index (TWI), (**h**) drainage density (Dd), (**i**) Distance from river, (**j**) stream power index (SPI), and (**k**) rainfall

Elevation is a critical factor in flood risk modeling, as it directly influences the flow of water during flood events. Water generally follows the path of least resistance, typically along the steepest descent^54^. In this study, elevation data were derived from the Digital Elevation Model (DEM) obtained from ALOS-PALSAR. It was downloaded from the Alaska Satellite Facility (ASF) website (https://www.asf.alaska.edu) at a spatial resolution of 12.5 m. This dataset was used to generate the elevation model of the Choke Mountain Watershed (Fig. 3a).

Using elevation data, identify flood-prone areas, estimate the extent and severity of potential flooding, and assess the likely impacts on infrastructure, communities, and ecosystems. Accurate elevation data are essential for developing effective flood risk management strategies. Thus, elevation is a fundamental parameter in flood susceptibility assessment^54^. Low-lying or flat areas are generally more susceptible to flooding due to slower water drainage and accumulation. Understanding the region’s topography and related geomorphological features is therefore vital for assessing flood vulnerability. Areas at lower elevations tend to experience higher flood risk^55^. Maps were created using ArcGIS software (version 10.4; Esri, Redlands, CA, USA; https://www.esri.com*).*

#### Slope

Slope is another key parameter in flood modeling, as it affects both the velocity and direction of surface water flow. Slope refers to the steepness or inclination of the terrain and is commonly derived from DEM data. Steeper slopes typically result in faster runoff and greater erosion potential, while flatter areas may experience slower drainage and increased water accumulation^1^. However, flood risk is influenced by additional factors such as land use, vegetation cover, and soil characteristics^54^. In some contexts, gentle slopes can extend the time available for infiltration, but they can also lead to a larger volume of surface runoff entering drainage systems, thereby increasing flood risk^54^. In this study, a slope map was generated using ArcGIS 10.4 software https://www.esri.com*)* from the DEM. The slope data were then classified into five categories to facilitate flood risk analysis (Fig. 3b).

#### Aspect

Aspect is a key factor influencing the direction of water flow and related hydrological processes^54^. It refers to the compass direction that a slope faces and plays a significant role in determining microclimatic conditions, soil moisture distribution, vegetation patterns, and overall watershed behavior^54^. Due to the impact of aspect on sunlight, exposure, and evapotranspiration rates, shady (north-facing) slopes tend to retain more moisture and experience less evaporation compared to sunny (south-facing) slopes. This can reduce the likelihood of waterlogging in shaded areas^1^. In this study, the aspect was derived from DEM data and categorized into five classes, each representing a specific cardinal or intercardinal direction. The distribution of flood-affected pixels was found to be relatively uniform across all aspect categories (Fig. 3c), suggesting that while aspect influences micro-conditions, its direct effect on flood susceptibility may be secondary to other topographic or hydrologic variables. Maps were created using ArcGIS software (version 10.4; Esri, Redlands, CA, USA; https://www.esri.com*).*

#### Plan curvature

Plan curvature, also known as horizontal curvature, describes the curvature of the terrain surface perpendicular to the slope direction. It reflects the ability of the landscape to converge or diverge surface water flow, thereby influencing water accumulation and distribution patterns^27^. In areas with a high positive curvature, the land surface is convex, which slopes upward in all directions. This leads to faster water flow and an increased risk of erosion. Conversely, areas with high negative curvatures are concave, so the land surface slopes downward, leading to slower water flow and increased risk of ponding. Therefore, the likelihood of flooding is generally inversely related to curvature—lower or more concave curvatures are associated with higher flood risk. In this study, plan curvature was derived from high-resolution elevation data using the ArcGIS software (version 10.4; Esri, Redlands, CA, USA; https://www.esri.com*).* The resulting curvature map helps to identify terrain features that enhance or impede surface water accumulation (Fig. 3d).

#### Land use/land cover (LULC)

Land use and land cover are among the most influential factors in flood modeling^56^. LULC plays a crucial role in regulating hydrological and geomorphological processes by directly or indirectly affecting evapotranspiration, infiltration, surface runoff, and sediment transport. Land cover changes, particularly urbanization, can greatly raise surface impermeability, which improves runoff and raises the danger of flooding. Ganjirad and Delavar^56^ demonstrated a strong correlation between flood-prone areas and land use changes, particularly in regions undergoing rapid urban expansion. In this study, the land use and land cover types were classified into six categories: agriculture, barren land, forest, pastoral land, urban areas, and water bodies. Classification was carried out using ArcGIS software (version 10.4; Esri, Redlands, CA, USA; https://www.esri.com*)* (Fig. 3e).

#### Soil types

Soil type is a fundamental factor in flood modeling, as it affects the soil’s ability to absorb water, regulate infiltration, and generate runoff. Different soil textures and compositions influence groundwater recharge rates and surface water movement, making soil data critical for flood hazard assessments^26^. Soil maps can identify areas with high runoff potential or limited infiltration capacity. When combined with other topographic and land use data, such as slope and LULC, soil information improves the accuracy of flood susceptibility modeling^54^. Furthermore, identifying soil types helps in planning mitigation strategies such as rainwater harvesting, green infrastructure, and soil conservation practices to reduce flood risk. Clay, sandy loam, and loam are among the soil types examined in the present study (Fig. 3f). Maps were created using ArcGIS software (version 10.4; Esri, Redlands, CA, USA; https://www.esri.com*).*

#### Topographic wetness index (TWI)

The Topographic Wetness Index (TWI) is a quantitative measure used to estimate spatial variations in soil moisture and potential water accumulation across a landscape. It relates the local upslope contributing area to the slope, reflecting the influence of topography on water movement and accumulation^57^. TWI is computed using Eq. 1.

1 $$TWI = \ln \cdot (\frac{{As}}{{\tan \beta }})$$

Where As is the upslope contributing area per unit contour length, and β is the local slope angle.

Higher TWI values indicate areas that are more likely to be saturated or accumulate water, while lower values suggest better drainage. This makes TWI a vital parameter in identifying zones with high runoff potential and flood risk. In this study, TWI values were derived from the DEM using standard hydrological analysis tools^58^, and the spatial distribution of TWI is presented in Fig. 3g. Maps were created using ArcGIS software (version 10.4; Esri, Redlands, CA, USA; https://www.esri.com*).*

#### Drainage density

Drainage density is a key parameter in flood modeling, as it reflects the extent of drainage networks within a watershed and significantly influences surface runoff and flood generation. It is defined as the total length of all streams and rivers in a basin divided by the basin’s total area^59^. GIS and hydrological modeling tools utilize drainage density data to simulate flood flow, delineate flood-prone zones, and assess the severity and spatial extent of potential flooding. This factor is especially useful for infrastructure planning, land-use regulation, and hazard mitigation^60^. A higher drainage density generally corresponds to a higher surface runoff rate and thus a greater likelihood of flooding.

In the current study, drainage density was calculated using a Digital Elevation Model (DEM) in ArcGIS software (version 10.4; Esri, Redlands, CA, USA; https://www.esri.com*).* The resulting drainage density map (Fig. 3h) provides valuable insight for flood risk assessment and supports effective flood management planning^55,60^.

#### Distance from river

The distance from rivers, streams, and canals is another important variable in flood risk modeling. Areas located closer to major drainage channels are typically more vulnerable to flooding due to direct exposure to runoff and potential overflow during high rainfall events^1,53,54^. This parameter plays a significant role in flood susceptibility mapping by indicating how proximity to water bodies affects exposure to flood hazards. Poorly drained areas can lead to water accumulation and increased flood risk, especially when runoff exceeds the natural or engineered drainage capacity.

In this study, the Euclidean Distance tool in ArcGIS software (version 10.4; Esri, Redlands, CA, USA; https://www.esri.com*)* was used to generate a spatial layer representing the distance from river features within the study area (Fig. 3i). Incorporating this parameter into flood models supports improved land-use planning, infrastructure design, and the development of comprehensive flood mitigation strategies.

#### Stream power index (SPI)

The Stream Power Index (SPI) is widely used in flood hazard modeling to estimate the erosive power of surface runoff and channelized flow. It is a function of both drainage area and slope and quantifies the potential energy available to move water and sediment in a watershed^54^. SPI is particularly effective in identifying areas with a high risk of erosion and flood-related damage, making it a valuable metric for both flood prediction and landscape management. It measures the intensity of flow accumulation and the capacity of the water to erode soil and reshape terrain.

In this study, SPI was calculated using DEM-derived data in ArcGIS software (version 10.4; Esri, Redlands, CA, USA; https://www.esri.com*).* SPI can be expressed as Eq. 2^26^.

2 $$SPI = As*\tan (\beta )$$

Where:

- As = upslope contributing area (flow accumulation),
- Tan (β) = local slope gradient.

The resulting SPI map (Fig. 3j) highlights zones of potentially high erosion and flow energy, thereby contributing to a comprehensive flood risk assessment.

#### Rainfall

Precipitation is a primary driver of flood events and plays a vital role in flood risk modeling, as it directly contributes to increased surface runoff and the overflow of rivers and water bodies^56^. Intense or prolonged rainfall can overwhelm natural and artificial drainage systems, significantly increasing the severity and spatial extent of flooding^56,59^.

Accurate precipitation data are essential for identifying flood-prone areas and understanding hydrological responses to rainfall. Rainfall information is typically collected using instruments such as rain gauges, weather radar, and satellite sensors. This data can then be processed uaing ArcGIS software (version 10.4; Esri, Redlands, CA, USA; https://www.esri.com*)* to support spatial flood analysis and model potential flood scenarios^54^. When integrated with other environmental variables—such as topography, land cover, and soil type—precipitation data enhances the accuracy of flood prediction models, supports the development of early warning systems, and informs emergency preparedness and mitigation strategies^56^.

In this study, rainfall data were acquired from four meteorological stations located within the Chokw watershed. The kriging interpolation method was applied using ArcGIS software (version 10.4; Esri, Redlands, CA, USA; https://www.esri.com*)* to create spatial rainfall distribution maps^59^. The resulting precipitation map is shown in Fig. 3k.

### Machine learning (ML) based flood susceptibility mapping

The application of machine learning (ML) algorithms has significantly enhanced the precision and efficiency of flood susceptibility mapping by capturing complex, nonlinear relationships among environmental, hydrological, and anthropogenic factors^51,61^.

The selection of these algorithms was guided by their proven robustness, high predictive accuracy, and ability to manage nonlinear and multicollinear relationships among topographic and hydrological predictors. Ensemble learning techniques such as Random Forest (RF), Gradient Boosting (GB), and Extreme Gradient Boosting (XGBoost) combine multiple weak learners (decision trees) to improve model generalization and reduce overfitting—issues commonly encountered in traditional single-model approaches such as logistic regression or classification and regression trees (CART)^4,26,45^. Numerous recent studies have also confirmed their superior performance in flood mapping, offering higher classification accuracy and better generalization across diverse environmental conditions^4,50,52^.

All causative factors used for modeling were prepared in a GIS environment. Data preprocessing and feature generation were performed using ArcGIS software (version 10.4; Esri, Redlands, CA, USA; https://www.esri.com*)*, including raster reclassification, normalization, and extraction of factor values. Remote sensing (RS) data, such as Sentinel imagery, were processed in the Sentinel Application Platform (SNAP 7.0). Machine learning model development and analysis were implemented in Python 3.9, employing the scikit-learn library (v1.1.3) for model building and evaluation. The modeling process consisted of three major stages: (1) data preparation and conditioning factor selection, (2) machine learning model training and validation, and (3) flood susceptibility map generation. The dataset was divided into 70% for training and 30% for validation, using the train_test_split function in scikit-learn. Each conditioning factor was standardized to a uniform spatial resolution (30 × 30 m) and normalized to the 0–1 range prior to modeling. This standardization prevents features with large numeric ranges (e.g., elevation, rainfall) from dominating those with smaller ranges (e.g., curvature, TWI) in the learning process.

#### Random forest (RF)

The Random Forest (RF) model is widely recognized in hydrological modeling for its resilience to overfitting, automatic handling of missing data, and robustness to outliers^52,62^. It is a widely used machine learning technique that has shown strong performance in flood susceptibility modeling and prediction^63^. For classification tasks, the final prediction is based on the majority vote of all decision trees, whereas for regression problems, the average of all individual tree predictions is used.

Each decision tree within the forest is constructed from a random subset of the training data and a random selection of input features, which introduces diversity among trees and enhances the model’s robustness^63^.

One of the primary advantages of Random Forest is its high training speed and its ability to handle multicollinearity and nonlinear relationships among input features. Moreover, RF is relatively resilient to missing data and imbalanced datasets, maintaining strong predictive accuracy even in challenging data conditions^26,64^.

These characteristics make RF particularly suitable for flood susceptibility modeling, where environmental factors often interact in complex and nonlinear ways. Additionally, RF can provide insights into the relative importance of each input variable, which is useful for understanding the most influential flood-inducing factors.

#### Gradient boosting (GB) model

The Gradient Boosting (GB) Model is a powerful ensemble machine learning algorithm that has demonstrated high performance in both classification and regression tasks, particularly on structured (tabular) datasets^29^. Gradient Boosting has been used extensively in flood hazard zoning (FHZ) because of its capacity to manage intricate linkages and produce precise forecasts.

Gradient Boosting operates as an additive model, where decision trees are built sequentially. Each new tree attempts to correct the errors made by the previous ensemble of trees by fitting to the negative gradient of the loss function, which represents the direction of steepest descent^29^. This iterative error-correcting process allows GB to minimize prediction error effectively and improve model accuracy at each stage.

Gradient Boosting develops trees in a dependent sequence, where each succeeding tree is trained to lower the residual errors from the previous one, in contrast to Random Forest (RF), which builds trees individually and aggregates their outputs^63^. This stage-wise approach continues until a stopping criterion is met, such as reaching a specified number of trees or achieving minimal improvement in performance.

The Gradient Boosting framework was used in this study to analyze flood susceptibility because it can reduce bias and variation and provides strong generalization capabilities even when complicated and nonlinear data linkages are present^63^.

#### Extreme gradient boosting (XGBoost)

Extreme Gradient Boosting (XGBoost) is an advanced machine-learning algorithm that has gained significant popularity in solving regression, classification, and ranking problems. It builds on the traditional gradient boosting framework, enhancing it with advanced regularization, parallel computation, and efficient handling of missing data^29,51,63^.

The Extreme Gradient Boosting (XGBoost) model, an optimized version of GB, integrates regularization terms into its objective function to reduce overfitting, enhance computational efficiency, and improve interpretability^52,54^. XGBoost has shown outstanding performance in numerous environmental modeling applications, including flood susceptibility mapping^65,66^. Comparative studies^4,52,65^ have confirmed that RF and XGBoost outperform conventional algorithms such as logistic regression, k-nearest neighbor (KNN), or support vector machines (SVM) in delineating flood-prone zones with higher AUC accuracy.

### Hyper-parameter optimization

Hyperparameter tuning plays a critical role in maximizing the predictive accuracy and generalization of machine learning models^52,67^. In this study, model-specific hyperparameters were optimized using the GridSearchCV function in the scikit-learn library^68^. This approach systematically explores predefined parameter combinations, performs cross-validation for each, and identifies the combination that achieves the highest validation accuracy. The optimization process was independently conducted for each of the three models— Random Forest, Gradient Boosting, and Extreme Gradient Boosting—using a 5-fold cross-validation scheme. Table 3 summarizes the tuned hyperparameters and their search ranges. The optimal hyperparameter values were selected based on the model performance (AUC and performance metrics) in the validation data.

To evaluate model robustness and prevent overfitting, a two-stage validation strategy was implemented. First, the dataset was divided into 70% training and 30% testing subsets using the train_test_split function in scikit-learn. Then, a 5-fold cross-validation was applied within the training subset during hyperparameter tuning to ensure model stability and generalization capability. In addition to this systematic validation framework, model-specific strategies were incorporated to further minimize overfitting. For the Random Forest (RF) model, out-of-bag (OOB) error estimation was used as an internal validation mechanism, and the number of features considered at each split was limited to improve generalization. In the Gradient Boosting (GB) model, overfitting was controlled by employing a small learning rate (0.02) and a moderate tree depth (max_depth = 5). The Extreme Gradient Boosting (XGBoost) model incorporated both L1 (α) and L2 (λ) regularization terms, constrained tree depth (max_depth = 6), and applied shrinkage (learning_rate = 0.1) to penalize excessive model complexity. The integration of rigorous hyperparameter tuning, multi-level validation, and targeted regularization strategies ensured that the developed models achieved stable, reliable, and generalized performance in flood susceptibility prediction, effectively minimizing bias and variance. All computations were performed on a workstation equipped with an Intel^®^ Core™ i7-10700 CPU @ 2.90 GHz and 16 GB RAM, using Python 3.9 and scikit-learn 1.2.2 libraries on Windows 10.

**Table 3:** Tuned hyper-parameters.

Model name	Tune parameter
RF	n_estimators = 200, max_depth = None, min_samples_split = 2, criterion Gini
GB	n_estimators = 250, learning_rate = 0.01, max_depth = 5, criterion mse
XGBoost	eta = 0.3, max_depth = 6, gamma = 0.01, subsample = 1, validate_parameters = TRUE, niter = 200, learning_rate = 0.1, min_child_weight = 0.05

### Model validation and performance evaluation

Accurate validation of flood susceptibility models is essential to ensure their predictive reliability and practical applicability. In this study, a combination of statistical metrics was employed to comprehensively evaluate model performance. The key indicators included the Area under the Receiver Operating Characteristic Curve (AUC-ROC), Positive Predictive Value (PPV), and Negative Predictive Value (NPV)^26,54,58^.

The AUC quantifies the model’s ability to distinguish between flooded and non-flooded areas, providing a single scalar value that reflects overall classification performance across all possible thresholds. An AUC value close to 1.0 indicates excellent discrimination capability, whereas a value near 0.5 suggests performance comparable to random chance. The Receiver Operating Characteristic (ROC) curve, which plots sensitivity (true positive rate) against 1 - specificity (false positive rate), offers a visual representation of this trade-off and allows for comparative evaluation of model accuracy.

In addition to classification metrics, several regression-based statistical indicators were used to evaluate the models’ predictive precision. These included the coefficient of determination (R²), root mean square error (RMSE), mean absolute error (MAE), and mean squared error (MSE). These indices are widely recognized as key performance measures in flood modeling and other environmental prediction studies due to their ability to quantify the goodness-of-fit and predictive deviation of models. Collectively, the use of AUC, R², RMSE, MAE, and MSE provided a robust framework for assessing the accuracy, consistency, and generalization ability of the applied machine learning models^26,54,63,64^.

3 $$R^{2} = 1 - \frac{{\sum\nolimits_{{i - 1}}^{n} {(p - \alpha )^{2} } }}{{\sum\nolimits_{{i - 1}}^{n} {(p - \alpha )} ^{2} }}$$

4 $$RMSE = \sqrt {\frac{1}{n}\sum\nolimits_{{i = 1}}^{n} {[(p - \alpha )]^{2} } }$$

5 $$MAE = \frac{1}{n}\sum\nolimits_{{i = 1}}^{n} {(p - \alpha )^{2} }$$

Where α is the actual value, ἀ is the mean of the actual values, p is the predicted value of the model, and n indicates the number of observations.

### Feature selection criteria

Selecting appropriate input features is a critical step in developing efficient and accurate machine-learning (ML) models for flood-susceptibility mapping. The inclusion of relevant, high-quality features enhances predictive performance, whereas irrelevant or redundant variables may introduce noise, increase computational cost, and degrade model accuracy^26^. To identify influential features and eliminate non-contributing variables, several statistical techniques are employed. One commonly used method is the Information Gain Ratio (InGR), which quantifies the contribution of each feature to the prediction of the target variable. A higher InGR value indicates a greater degree of influence, making it a valuable criterion for prioritizing features^54^. Similarly, several published articles suggest the application of the Variable Inflation Factor (VIF) and Tolerance (TOL) for the evaluation of collinearity dependence. It is recommended that a model to be free from the multicollinearity tolerance should have a value greater than 0.1, and VIF should have a value less than 10^26^.

### Uncertainty and sensitivity analysis

To assess the robustness and reliability of the flood susceptibility models, both uncertainty analysis (UA) and sensitivity analysis (SA) were performed. UA quantifies how uncertainties in input parameters propagate to model outputs, while SA identifies which parameters contribute most to output variability^69^.

In this study, the SHapley Additive exPlanations (SHAP) method^70^ was used to interpret the contribution of individual predictors in the model. SHAP values tell us how much each feature affects the predictions made by machine learning models. After training the machine learning model, SHAP values were computed to assess the impact of each feature on individual predictions. SHAP values indicate a feature contributes to the model’s output: i.e., positive values increase the prediction (e.g., higher flood susceptibility). Negative values decrease the prediction (e.g., lower flood susceptibility). The magnitude reflects the strength of the feature’s influence. Together, the MC-based Sobol’ sensitivity and SHAP interpretability analyses provided complementary insights—quantifying uncertainty propagation and explaining factor-level influences—thereby strengthening the confidence in the model’s predictive robustness and interpretability.

## Results

### Variable importance

Before producing the flood-susceptibility maps (FSMs), the relative importance of each flood-conditioning factor was quantified to identify the most influential predictors and verify the absence of redundancy. The Information Gain Ratio (IGR) was first applied to evaluate each factor’s contribution to flood occurrence, followed by multicollinearity diagnostics using the Variance Inflation Factor (VIF) and Tolerance (TOL).

**Fig. 4:**
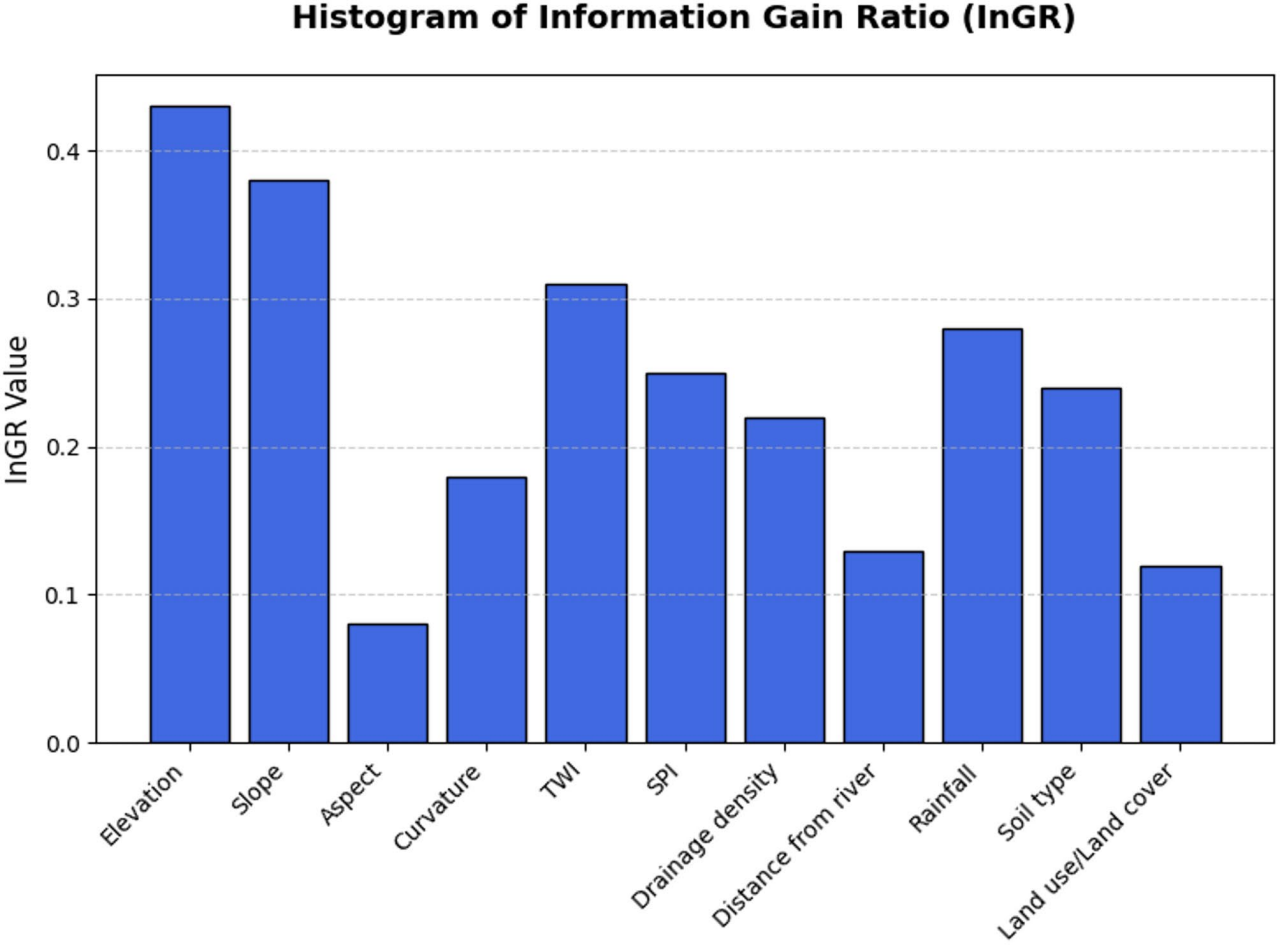
Information gain ratio (InGR)

**Fig. 5:**
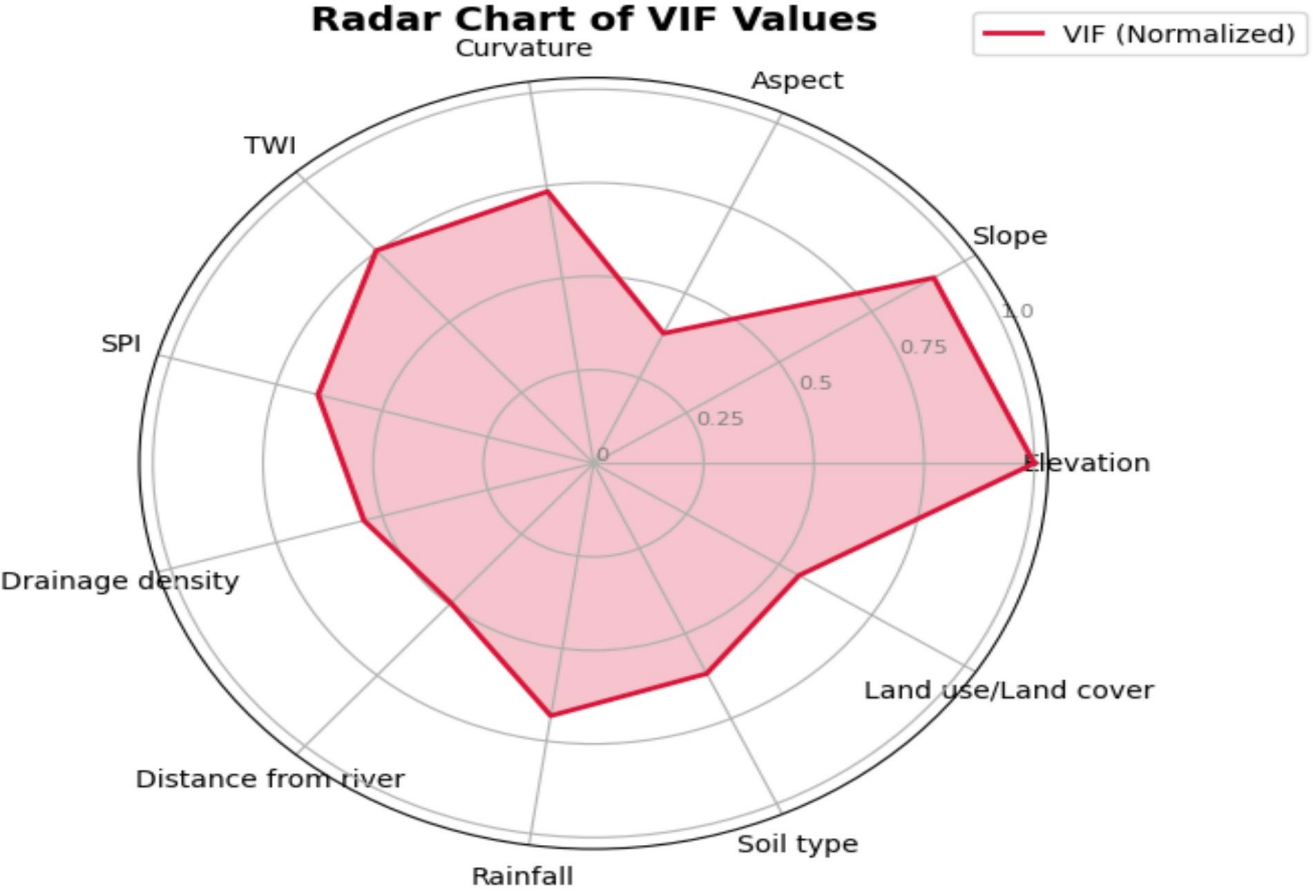
Radar chart of VIF and TOL

The IGR analysis (Fig. 4) shows that elevation is the most critical factor, followed by slope, topographic wetness index (TWI), rainfall, SPI, soil, drainage density, and curvature. These results align with previous research highlighting the dominant role of topographic variables in controlling flood susceptibility^32,33^. Conversely, distance from river, land use/land cover, and aspect are comparatively of lower importance. Similar findings were reported by^28,31,71^. A multicollinearity test was performed using VIF and TOL values to make sure all input components were statistically independent (Table 4). The obtained VIF values ranged from 1.02 to 2.67, while TOL values ranged from 0.37 to 0.98, all well within the accepted thresholds (VIF < 10, TOL > 0.1)^4,26,29^. These results confirm that the eleven selected flood-conditioning factors are free from multicollinearity and thus suitable for reliable model development. Furthermore, Fig. 5 shows the radar chart of VIF and TOL.

**Table 4:** Multicollinearity analysis of the flood conditioning factors.

No.	Conditioning factor	VIF	TOL
1	Elevation	2.67	0.37
2	Slope	2.45	0.41
3	Aspect	1.02	0.98
4	Curvature	1.96	0.51
5	TWI	2.01	0.50
6	SPI	1.74	0.57
7	Drainage density	1.45	0.69
8	Distance from river	1.32	0.76
9	Rainfall	1.82	0.55
10	Soil type	1.65	0.61
11	Land use/Land cover	1.48	0.68

**Fig. 6:**
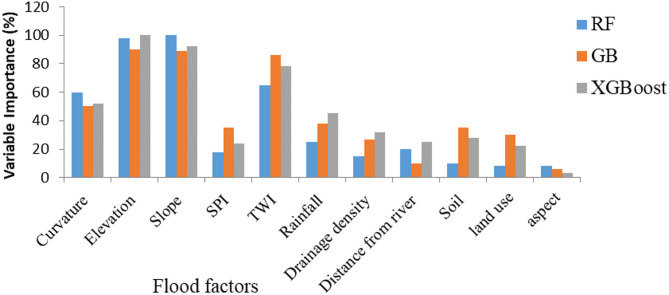
Variable importance of the used models

The relative importance of flood-conditioning factors varied slightly among the three ensemble models (RF, GB, and XGBoost), but all exhibited a consistent pattern dominated by topographic controls (Fig. 6).

In the Random Forest (RF) model, elevation, slope, and topographic wetness index (TWI) were identified as the most influential predictors. Variables with moderate importance included curvature, rainfall, stream power index (SPI), and drainage density. In contrast, soil type, land use/land cover (LULC), and aspect contributed minimally to flood susceptibility prediction.

Similarly, the Gradient Boosting (GB) model highlighted elevation, slope, and TWI as the leading factors influencing flood occurrence. Medium to lower influence was observed for curvature, SPI, soil type, drainage density, LULC, and rainfall. Factors such as distance from river and aspect had negligible predictive power in the GB model.

In the Extreme Gradient Boosting (XGBoost) model, elevation, slope, and TWI again ranked as the three most important variables, confirming the robustness of these topographic indicators across algorithms. Variables with moderate to lesser influence included curvature, rainfall, drainage density, soil type, LULC, SPI, distance from river, and aspect.

Overall, all models consistently identified elevation, slope, and TWI as the dominant factors controlling flood susceptibility, underscoring the critical role of terrain-driven hydrological processes in the Choke Watershed. The relatively minor importance of aspect and rainfall suggests that short-duration, high-intensity precipitation events primarily trigger flooding through rapid runoff accumulation rather than prolonged rainfall or orientation-related effects, consistent with findings from previous studies^4,26,45,48^.

### Flood susceptibility mapping

 The analysis of flood susceptibility using multiple machine learning (ML) models revealed varying spatial distributions and classification outputs. These variations reflect the differences in how each model interprets the degree of flood susceptibility within the study area, ultimately aiding in the identification and prediction of flood-prone zones.

**Fig. 7:**
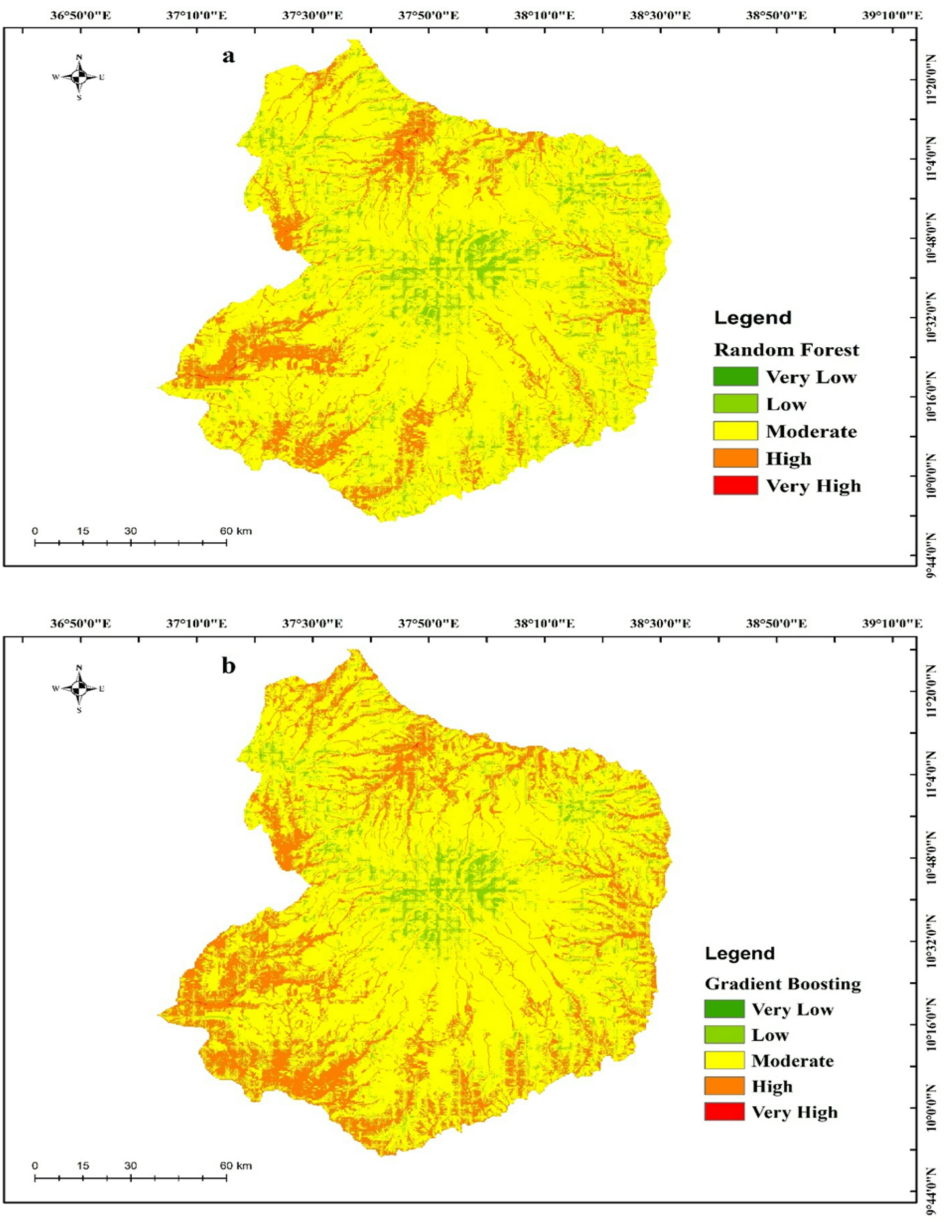
Flood susceptibility mapping with: (**a**) RF, (**b**) GB, and (**c**) XGBoost

Flood Susceptibility Maps (FSMs) were generated using three machine learning algorithms—Random Forest (RF), Gradient Boosting (GB) Model, and Extreme Gradient Boosting (XGBoost)—for the Choke Watershed of Ethiopia. In the literature, there are numerous ways of classifying the produced susceptibility map; nevertheless, the natural break classification approach is commonly used and is useful for comprehending findings at class borders^8,36^. The breaks are picked based on the values, where classes suggest the best groups. Each pixel in the susceptibility maps was categorized into one of five risk groups (very high, high, moderate, low, and very low) in order to compare the susceptibility levels. The resulting FSMs for each machine learning approach are shown in Fig. 7. These maps show the likelihood of flooding in a given place based on the associated conditioning factors. According to the RF susceptibility Map, the very low, low, moderate, high, and very high classes cover, respectively, 3.15%, 6.15%, 70.3%, 12.2%, and 8.2%. Similarly, the very low, low, moderate, high, and very high percentage classes of the GB model were estimated as 5.23%, 6.33%, 59.42%, 21.12, and 7.90%, respectively. In addition, the flood-predicting percentage classes of the XGBoost, were estimated as 2.2%, 4.2%, 67.05%, 20.02%, and 6.53% of the total surface area correspond to the very low, low, moderate, high, and very high classes, respectively.

One of the most significant findings was that many areas in the northern and southwestern parts of the study area were located in high- and very high-risk zones. These zones are topographically characterized by steep gradients between elevated and low-lying terrain, which intensify water accumulation during heavy rainfall events. The steep slopes also promote bank undercutting, enhancing the ease of overland flow and erosion. This is primarily because of their proximity to the mountains, which contribute to flooding through several mechanisms. Intense rainfall events generate large volumes of runoff, and landslides can block streams or create new ones. Steep mountain slopes can increase the speed and force of water as it flows downhill, potentially causing significant damage to property and infrastructure.

### Evaluating the performance of different machine learning (ML) algorithms

To assess the predictive performance of the applied machine learning (ML) algorithms, Receiver Operating Characteristic (ROC) curve analysis was conducted. The ROC curves were used to calculate the Area under the Curve (AUC) values, which reflect each model’s ability to distinguish between flood-prone and non-flood-prone areas. AUC values closer to 1.0 indicate higher model accuracy.

**Fig. 8:**
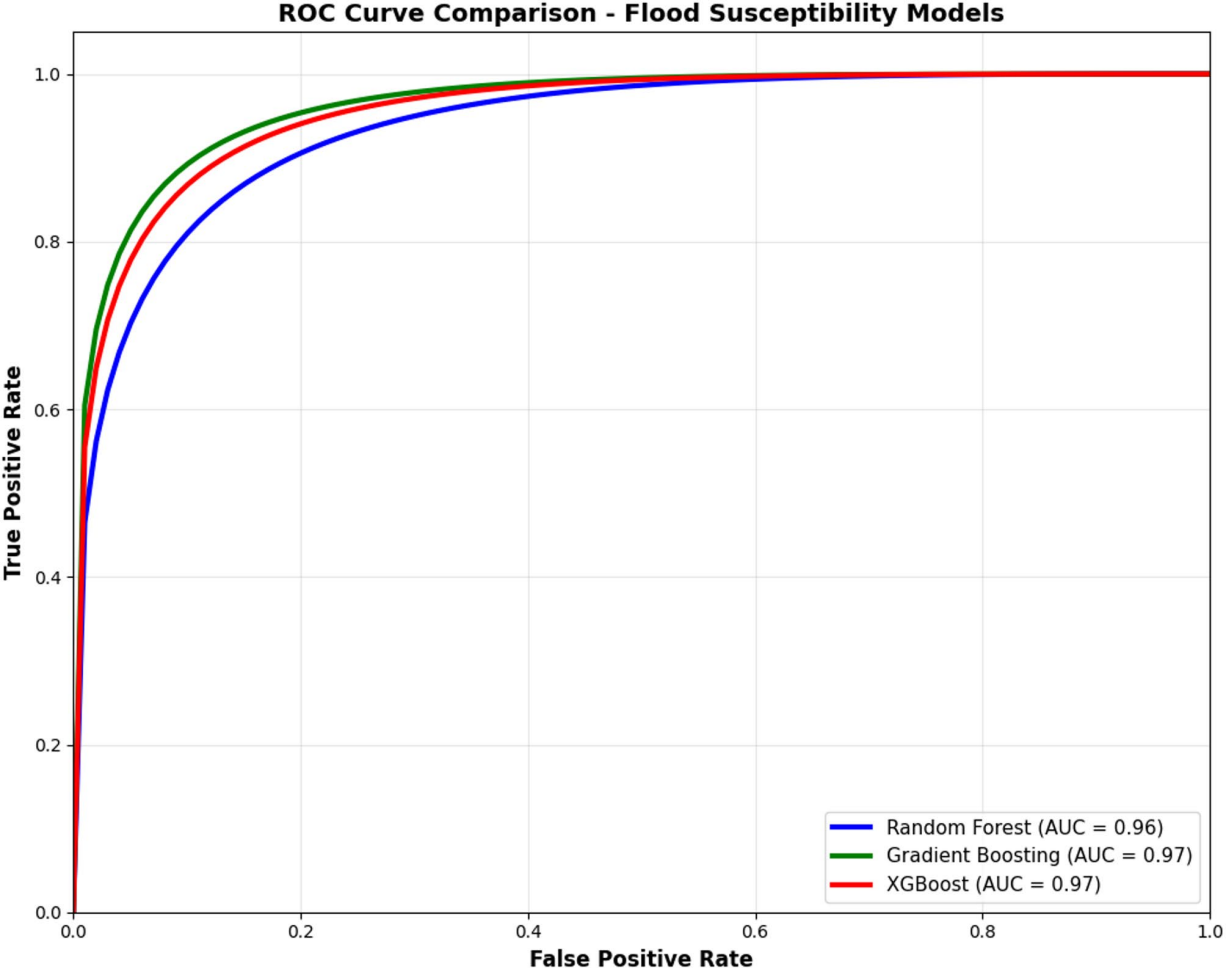
ROC curve of the GB, XGBoost, and RF models validation

Among the tested algorithms, Gradient Boosting (GB) and Extreme Gradient Boosting (XGBoost) achieved the highest test accuracy (AUC = 0.97), followed closely by Random Forest (RF) with an AUC of 0.96. These findings highlight the superior performance of tree-based ensemble methods in flood susceptibility mapping (FSM). Figure 8 shows the ROC curve of Gradient Boosting (GB), Extreme Gradient Boosting (XGBoost), and Random Forest (RF) respectively.

Besides the ROC curve method, other performance metrics – RMSE, MAE, AUC, and R2 – were used to evaluate the models’ performances. As shown in Table 5, the best fitting model was GB, with RMSE = 0.1241, MAE = 0.0716, AUC = 0.97, and R^2^ = 0.9132, followed by XGBoost, with RMSE = 0.1535, MAE = 0.0440, AUC = 0.97, and R^2^ = 0.8932.

**Table 5:** Performance parameters for different machine learning models.

Model	RMSE	MAE	AUC	*R* ^2^
RF	0.2243	0.1245	0.96	0.8712
XGBoost	0.1535	0.0440	0.97	0.8932
GB	0.1241	0.0716	0.97	0.9132

The effectiveness of GB and XGBoost in FSM is corroborated by previous studies. For instance, Addis^34^used the FR method for flood risk mapping in the upper Abay River Basin,​ Ethiopiaand reported an AUC of 0.83, substantially lower than the 0.97 achieved in the present study. Similarly, Soltani, et al^3^. developed an FSM model using the NDVI index and rainfall data, obtaining an AUC of 0.82, again indicating comparatively lower predictive accuracy.

In another study, Madhuri, et al.^72^ evaluated five machine learning algorithms—Logistic Regression, Support Vector Machine (SVM), K-Nearest Neighbors (KNN), Adaptive Boosting (AdaBoost), and XGBoost—for the Greater Hyderabad Municipal Corporation (GHMC) in India. Their results showed that XGBoost outperformed the other models, achieving the highest mean AUC score of 0.83.

Several recent studies further support these findings. Sahin^73^ assessed XGBoost, SVM, RF, and logistic regression for FSM, concluding that XGBoost produced the most promising results. Similarly, Sahin^74^ evaluated Gradient Boosting Decision Trees (GBDT), RF, and CatBoost models and reported that Gradient Boosting outperformed the alternatives.

Furthermore, Saravanan and Abijith^58^ evaluated multiple machine learning models—including GBM, XGBoost, Rotation Forest (RTF), SVM, and Naive Bayes (NB)—for flood susceptibility mapping in northern coastal Tamil Nadu. Their results indicated that GB and XGBoost achieved the best performance, with AUCs of 0.92 and 0.91, respectively.

Overall, the findings of the present study are consistent with the existing literature, confirming that ensemble-based models—especially GB and XGBoost—are highly effective for flood susceptibility mapping.

### SHAP analysis

**Fig. 9:**
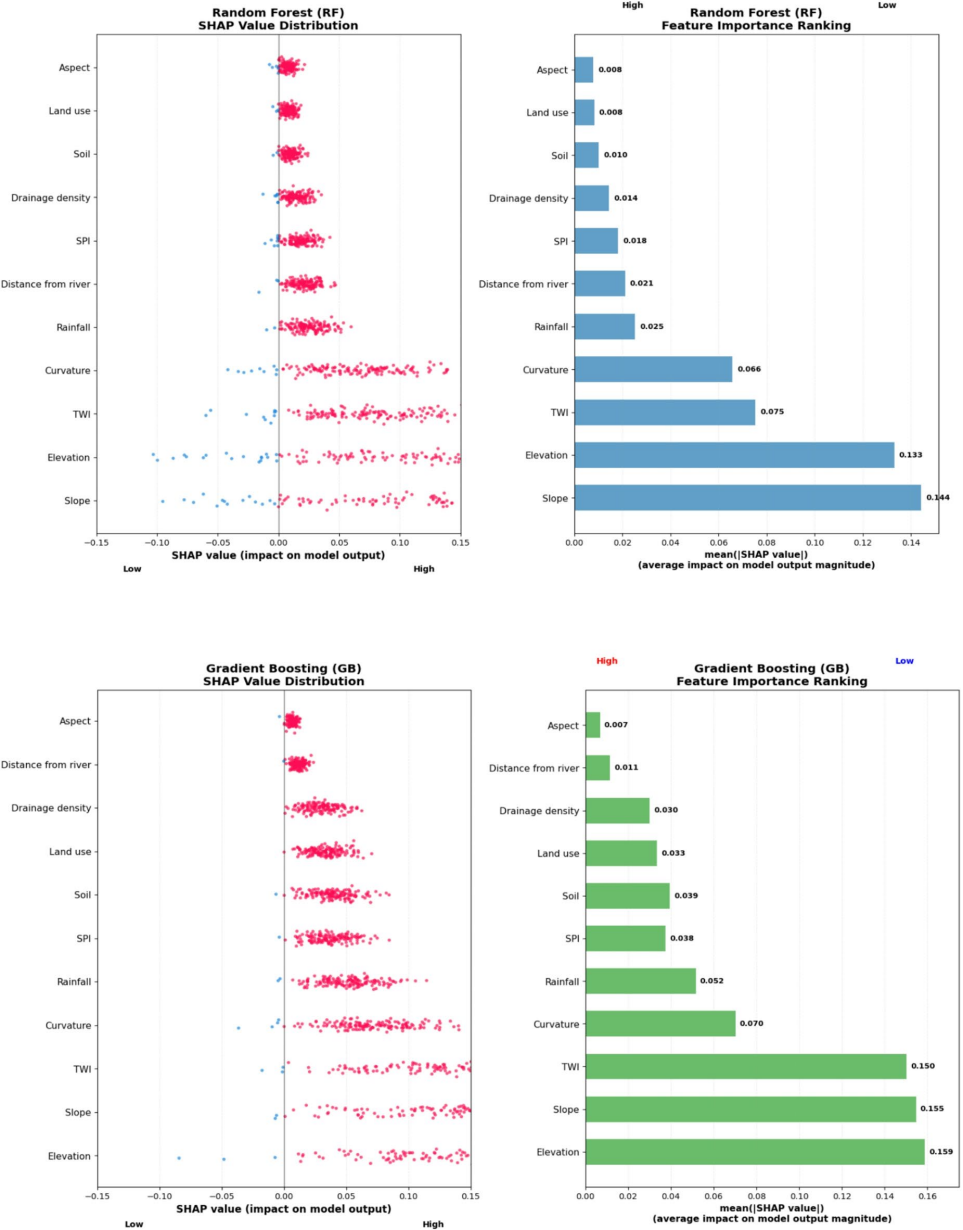
SHAP values for RF, GB, and XGBooset models.

Figure 9 shows SHAP summary plots depicting feature importance rankings for flood susceptibility modeling across three machine learning algorithms: Random Forest (RF), Gradient Boosting (GB), and eXtreme Gradient Boosting (XGBoost). The plots display SHAP values on the x-axis, representing the impact on model output, while the conditioning factors are arranged on the y-axis in descending order of importance.

According to the SHAP analysis, elevation and slope consistently emerged as the most influential factors across all models, though their relative importance varied between algorithms. In Random Forest, slope demonstrated the highest impact (SHAP value: 0.161), followed closely by elevation (0.151). Conversely, in both Gradient Boosting and XGBoost models, elevation ranked as the dominant factor (SHAP values: 0.159, and 0.150 respectively), with slope being the second most important feature.

TWI (Topographic Wetness Index) and curvature established themselves as consistently important secondary factors, consistently appearing among the top five influential variables across all three models. Rainfall demonstrated moderate but consistent contribution to flood prediction accuracy, maintaining stable importance rankings. The analysis revealed that topographic features (elevation and slope) and hydrological characteristics (TWI) collectively exerted the most significant influence on flood susceptibility predictions.

Among the least influential factors, aspect was consistently ranked as the least important variable across all models, followed by land use, soil characteristics, and drainage density. Distance from river and SPI (Stream Power Index) showed moderate contributions but ranked lower in the importance hierarchy. The remarkable consistency in feature importance rankings across all three machine learning algorithms reinforces the reliability of these findings and provides robust evidence for prioritizing these conditioning factors in flood susceptibility assessment and modeling efforts.

## Discussion

Floods remain among the most devastating natural disasters globally, affecting millions each year^63^. Enhancing flood susceptibility predictions is therefore vital for disaster risk reduction, early warning systems, and efficient resource allocation^4,45^.

Traditional flood mapping methods, relying on satellite imagery or ground-based observations, often suffer from limited spatial coverage and poor temporal resolution^63^. Currently, comparative analysis of standalone ML models. is very popular for flood susceptibility mapping due to its optimal accuracy, computational power, robust approach, execution time, and satisfactory performance^4,63^. Supervised ML models such as RF, GB, and XGBoost are particularly adept at processing large datasets and extracting complex non-linear patterns to delineate flood-prone zones^54^.

Feature importance analysis identified elevation, slope, Topographic Wetness Index (TWI), and SPI as the most influential variables in this study. This aligns with existing literature that highlights topographic and hydrologic characteristics as key drivers of flood susceptibility^26,58,61,75^.

Steep slopes, significantly reducing the absorption amount of the soil, accelerate the surface runoff induced by precipitation. Areas with a low slope are more likely to flood due to excess water stagnation, resulting in severe flooding. As a result, the slope plays a significant role in regulating surface runoff, infiltration, and water retention, affecting an area’s flood susceptibility^54,51,63^.

The Standardized Precipitation Index (SPI) was also found to be a key variable, indicating the level of soil moisture and the potential for downward water flow. Areas with lower SPI values are more likely to accumulate runoff, increasing flood risk. Similarly, Zhang, et al.^76^ also found that low-elevation, low-vegetation zones—often composed of coarse sediments like marl and sand—are particularly flood-prone.

The Topographic Wetness Index (TWI) plays a pivotal role in quantifying soil moisture distribution. Areas with higher TWI values showed a strong correlation with flood-prone zones, indicating that increased moisture content significantly elevates the likelihood of flooding^75^. Although rainfall is the primary triggering factor for flooding, its relative importance in this study was lower compared to topographic variables such as elevation, slope, and the Topographic Wetness Index (TWI). This outcome can be attributed to the relatively uniform spatial distribution of rainfall across the Choke Watershed, which limits its ability to differentiate localized flood-prone zones in the model. In contrast, topographic and hydrological factors exert a stronger control on surface runoff, flow accumulation, and water convergence, thereby having a greater influence on flood susceptibility patterns. Moreover, short-duration, high-intensity rainfall events—typical of the region’s convective storms—often generate flash floods that are highly sensitive to slope and drainage characteristics rather than total rainfall amount. In the Choke Watershed, rainfall primarily acts as a triggering mechanism, while the spatial variability of flood susceptibility is predominantly governed by terrain-driven runoff dynamics and land surface conditions. These findings are consistent with previous studies that also reported rainfall as a secondary or moderately important variable when topographic factors dominate hydrological responses in mountainous catchments^26,45,65^.

All three evaluated models achieved AUC-ROC values exceeding 0.90, indicating excellent classification performance. However, GB and XGBoost—both tree-based ensemble models—outperformed algorithms, consistent with prior studies^26,54,77^. Setianto, et al.^78^, in a study on flood susceptibility in the Musi River basin, Hyderabad, India, found that XGBoost outperformed logistic regression, support vector machine (SVM), K-nearest neighbors (KNN), and AdaBoost—aligning closely with the findings of this study. Similarly, Wang, et al.^79^ demonstrated that XGBoost outperformed traditional regression models in predicting mortality from moderate-to-severe traumatic brain injury. Demir and Şahin^80^ applied SVM, RF, and XGBoost for liquefaction prediction using a genetic algorithm-based feature selection method and reported that XGBoost provided the highest prediction accuracy, including in flash flood susceptibility modeling.

## Study limitations and future directions

Despite the promising results, this study has several limitations that should be acknowledged. Most notably, the absence of critical hydrological parameters—such as flood depth, river discharge, and flow velocity—restricts the model’s ability to simulate the physical dynamics of flooding accurately. Additionally, subjective choices made during the modeling process, including the selection of input features, classification thresholds, and training/testing data splits, may introduce bias or uncertainty. The reliance on a single performance metric, such as the Area under the Curve (AUC), although widely used, may not fully reflect model performance under all conditions or scenarios.

To address these challenges and enhance future flood susceptibility mapping efforts, several improvements are recommended. Integrating detailed hydrological data, including peak discharge and flood depth, would improve model realism and interpretability. Employing higher temporal resolution datasets—such as daily or hourly rainfall—could capture short-term flood events often missed by coarser temporal data. Standardizing the spatial resolution of input layers is also essential to minimize errors introduced by mismatched data scales. Furthermore, involving local stakeholders, such as government agencies and community members, in selecting and validating flood-related variables can help ensure that models are contextually relevant and grounded in local knowledge. Lastly, conducting comparative studies across multiple regions with varying climatic, geological, and land use conditions would help validate the robustness and generalizability of machine learning models for flood susceptibility mapping.

## Conclusions

This study applied three ensemble machine learning (ML) models—Random Forest (RF), Gradient Boosting (GB), and Extreme Gradient Boosting (XGBoost)—to generate a detailed flood susceptibility map of the Choke Watershed, Ethiopia. The comparative results revealed that the GB and XGBoost models achieved the highest predictive accuracy (AUC = 0.97), followed closely by the RF model (AUC = 0.96), demonstrating the strong capability of tree-based ensemble methods in capturing complex, nonlinear hydrological and topographic relationships.

Spatially, the analysis identified that approximately 6.5%–8.2% of the watershed is classified as highly flood-prone, with these zones predominantly located in the northern and southwestern parts of the Choke Watershed. These areas coincide with low-lying floodplains, gentle slopes, and zones of high surface runoff accumulation, reflecting the dominance of topographic and hydrological controls in shaping flood susceptibility. Conversely, central and high-elevation regions exhibited lower susceptibility levels due to enhanced drainage capacity and steep terrain.

From a management perspective, the results provide critical insights for local policymakers, planners, and disaster risk authorities. The susceptibility maps can serve as a foundation for prioritizing flood mitigation investments, guiding land-use planning, and informing early-warning and emergency preparedness programs. In particular, development restrictions in high-risk floodplains, reinforcement of drainage infrastructure, and the promotion of nature-based solutions (e.g., watershed restoration and reforestation) are recommended to reduce flood impacts.

Although the models performed robustly, some uncertainty remains due to limited availability of high-resolution rainfall and hydrodynamic data. Future studies should integrate hydraulic simulation outputs (e.g., flood depth and flow velocity) and updated climate projections to further refine the accuracy of susceptibility mapping.

Overall, this research underscores the potential of ensemble ML algorithms as effective and reliable tools for data-driven flood risk assessment, offering valuable guidance for sustainable watershed management and disaster resilience planning in data-scarce regions like the Choke Watershed.

## Data Availability

Datasets generated and/or analyzed during the current study are available from the corresponding author on reasonable request.
